# Case report: Treatment of severe phorate poisoning

**DOI:** 10.3389/ftox.2025.1581362

**Published:** 2025-05-09

**Authors:** Wang Kunlun, Yuan Jun, Xu Shiming, Shen Jie, Gao Mingqiang, Zhang Yuxia, Mo Weichun

**Affiliations:** ^1^ Center of Emergency and Intensive Care Unit, Research Center for Chemical Injury, Shanghai, China; ^2^ Emergency and Critical Medicine, Jinshan Hospital, Fudan University, Shanghai, China; ^3^ Emergency Department, Zhenyuan Yi Hani Lahu Autonomous County People’s Hospital, Puer, Yunnan, China; ^4^ Acute Poisoning Treatment Center of Zhenyuan County People’s Hospital, Puer, Yunnan, China

**Keywords:** organophosphate, phorate, poisoning, phoratoxon sulfoxide, phoratoxon sulfone, phorate sulfone, phorate sulfoxide, phoratoxon

## Abstract

**Background:**

Phorate is a highly toxic organophosphorus pesticide. Owing to its low cost and insecticidal potency, it is still widely used in parts of China, resulting in cases of occupational and life poisoning. This article presents the treatment process for phorate poisoning and monitoring the toxic metabolites terminology in the body.

**Case report:**

A 23-year-old male patient ingested about 300 mL (180 g) of 60% phorate emulsion 4 h before admission at our hospital. The patient had ingested over 300 times the lethal dose. During hospitalization, the patient’s levels of cholinesterase, phorate and its metabolites, atropine and pralidoxime chloride (PAM), were monitored. Phorate was quickly absorbed into the blood, producing five metabolites. Before hemoperfusion (HP), the concentration of phorate in the blood could not be detected. After the first HP we found five metabolites of phorate in the blood (phoratoxon sulfoxide,phoratoxon sulfone, phorate sulfone, phorate sulfoxide, and phoratoxon). In the follow-up treatment, the concentration of five metabolites gradually decreased. The concentration of the phorate sulfoxide and phorate sulfone rebounded with the suspension of HP, but that of the other metabolites did not rebound. It took 20 days for cholinesterase to recover. Treatment included multiple rounds of HP, atropinization, and reactivator of cholinesterase by PAM. The patient recovered after 34 days and was discharged from hospital.

**Conclusion:**

Phorate is oxidized and catalyzed into five metabolites, which cause the toxic effects. Phoratoxon sulfoxide has the highest concentration of these metabolites, followed by phoratoxon sulfone, phorate sulfone, phorate sulfoxide, and phoratoxon, respectively. HP treatment significantly lowered the serum levels of the toxic metabolites terminology. If HP treatment is interrupted, the serum levels of phorate sulfoxide and phorate sulfone tend to rise again. It takes a long time for cholinesterase to recover from severe phorate poisoning.

## Introduction

Organophosphorus pesticides (OPS) are the most widely used insecticides in China. They include dichlorvos, parathion (1605), phorate (3911), dimethoate, trichlorfon, and malathion (4049) (Kwong, 2002). Of these, parathion, and phorate are highly toxic varieties of organophosphorus pesticides (National Center for Biotechnology Information, 2025e; National Center for Biotechnology Information, 2025d). Once these compounds are ingested, the patient’s condition becomes critical, is difficult to treat, and the prognosis is poor. Phorate is widely applied in agriculture to control crop insect pests, including beans, corn, cotton, potatoes, radishes, sorghum, and beets (National Center for Biotechnology Information, 2025d). Owing to its toxicity to birds, fish, and reptiles, phorate is classified as a restricted pesticide. Phorate is highly volatile, and inhalation is the main route for poisoning people and animals (Kim et al., 2017).

In some cases, it has been used for self-poisoning (London et al., 2005; Mew et al., 2017; Gunnell et al., 2017; Eddleston et al., 2008). Phorate’s main mechanism of poisoning is via irreversible inhibition of acetylcholinesterase (Eddleston et al., 2008). Other toxicity mechanisms include DNA damage and cytotoxicity (Saquib et al., 2011; Saquib et al., 2012a; Saquib et al., 2012b; Sun et al., 2014; Saquib et al., 2019). Once absorbed, phorate is rapidly distributed in the body. It is then metabolized into various metabolites (McLeod et al., 1969), perpetuating its toxicity in the body and greatly complicating clinical treatment. There are few case reports on successful treatment (Khatiwada et al., 2012; Montana et al., 2021). Available reports mainly involve empirical treatment combined with routine treatment and HP. However, these reports lacked detailed and comprehensive monitoring of drug levels and metabolites in the blood and a thorough understanding of the metabolic processes and mechanisms of the toxicant (Khatiwada et al., 2012; Simonelli et al., 2022). Here, we report a severe case of phorate poisoning admitted to our hospital. Serum levels of phorate metabolites were carefully monitored, and their oxidation and catalytic metabolism were documented during acute phorate poisoning treatment. To the best of our knowledge, this is the first comprehensive description of the successful treatment of a severe case of phorate poisoning.

## Case report

**FIGURE 1:**
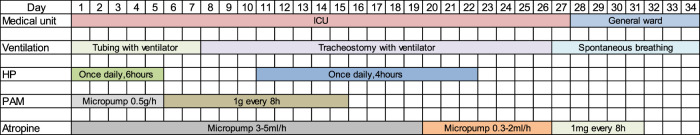
Schematic presentation of the clinical course.

A 23-year-old male patient ingested about 300 mL (180 g) of 60% phorate emulsion 4 h before admission at our hospital. Family members brought him to our hospital’s emergency department immediately after he was discovered. On hospital arrival, the patient exhibited deliriousness, restlessness, urinary incontinence, sweating, tears, dyspnea, but no fever. His blood pressure was 164/112 mmHg, heart rate was 132 times/min, SpO2 was 96%, bilateral pupil diameter was 0.2 cm, and the patient showed a light response. We gave him atropine with a maintenance pump, warm water for gastric lavage (Benson et al., 2013), PAM for intravenous drip treatment (Simonelli et al., 2022), and prepared for blood perfusion treat1ment. 15 min later, the patient experienced violent vomiting, which led to dyspneic and comatose, with a SpO2 of 81%. Blood gas analysis revealed a pH of 7.21, PaO2 of 53 mmHg, PaCO2 of 78 mmHg, base excess of −5.6 mmol/L, and lactate concentration of 8.7 mmol/L. Endotracheal intubation and ventilator-assisted ventilation were performed. The patient was moved into ICU after the first blood perfusion in the emergency room. Physical examination upon transfer into ICU revealed deep coma, GCS scale 4, Apache II score 22, bilateral pupil diameter of 0.4 cm, slow response to light, dry skin, thick breath sound in both lungs, non-obvious dry and wet rales, heart rate of 125 times/min, blood pressure of 98/65 mmHg, no swelling on both legs, low tension in limbs, and bilateral Babinski sign is absent. Blood gas analysis revealed a pH of 7.12, PaO2 of 182 mmHg, PaCO2 of 54 mmHg, base excess of −8.2 mmol/L, lactate concentration of 10.5 mmol/L, and serum cholinesterase at 268 U/L (Soreq and Seidman, 2001). The patient was continued on twice daily clear water gastric lavage for 4 days, continuous blood perfusion, PAM intravenous drip, atropine maintenance pump push and symptom-relieving treatment. Atropine treatment was administered using a continuous micropump until atropinize (pupil expands to a diameter of 0.4 cm, the heart rate is 100–120 beats per minute, the skin is dry, the face is flushed and the lung rale disappears). In the first 19 days, in order to maintain atropinization, the dosage of atropine was 3–5 mg per hour (about 70–120 mg/day). After the increase in cholinesterase, the dosage was gradually reduced. On the 27th day, the dosage was reduced to 1 mg every 8 h by subcutaneous injection. The total dosage of atropine during the course of the disease was about 1560 mg. During the initial 5 days post-admission, the dosage of PAM was 0.5 g per hour. On the sixth day, the concentration of PAM was significantly increased, and the inactivated cholinesterase was difficult to recover. The dosage was then reduced to 1.0 g intravenously every 8 h to prevent the newly produced cholinesterase from being inactivated again. The drug was discontinued on the 16th day.On the third day after admission, the patient’s consciousness improved, but he was restless and was therefore sedated with dexmedetomidine and midazolam. From the first to the fifth day of admission, HP was done for 6 hours, once daily (Wille et al., 2013; Worek et al., 2012). We employed the CVVHDF (Continuous Veno-Venous Hemodiafiltration) mode with post-dilution. The substitution volume was set at 1800 mL/h (the patient’s weight was 78 kg, approximately 23.1 mL/kg/h), and the dialysis volume was 1000 mL/h. The blood flow rate was initially set at 120 mL/min for the first 30 min and gradually increased to 180 mL/h. The transmembrane pressure (TMP) was maintained between 50–380 mmHg. Low-molecular-weight heparin (LMWH) was chosen as the anticoagulant, with an initial dose of 2,500 U and a maintenance dose of 250 U/h. Coagulation function was monitored at the third and sixth hours to maintain the activated partial thromboplastin time (APTT) between 1.5–2.5 times the normal value (35–60 s) (Sun, 2015).HP was stopped on the sixth to 10th day due to objection from the patient’s family. After explaining the benefits of HP, the family agreed to continue treatment. Blood was perfused for 4 h daily from the 11th to the 22nd. During the treatment, serum levels of cholinesterase, phorate, and its metabolites, atropine and PAM, were continuously monitored. On the eighth day after admission, cholinesterase levels remained between 200 and 300 U/L.The patient experienced psychiatric symptoms due to the administration of a large dose of atropine, rendering him unable to cooperate with treatment and agitated. To ensure treatment safety, sedative medication was required. However, high-dose medication could cause respiratory depression, and the patient had a pulmonary infection with poor sputum expectoration, so a tracheotomy was performed. (Hulse et al., 2014; Eddleston et al., 2004). Cholinesterase levels remained very low for 19 days after admission but steadily rose from the 20th day. Thus, sedatives were gradually withdrawn, and the patient began to regain consciousness. By the 23rd day, cholinesterase levels had reached 1,634 U/L, and blood perfusion was stopped. At the same time, a ventilator was gradually trained. On the 26th day, the tracheotomy was closed. On the 27th day, cholinesterase levels had reached 3592 U/L. Atropine was subcutaneously injected at 1 mg per 8 h, and the patient was transferred to the general ward for further treatment. By the 34th day, the patient recovered well, did not complain of discomfort, had no positive physical signs, and all tests had returned to normal. Cholinesterase levels had risen to 4,821 U/L. The levels of phorate and its metabolites in the serum were <10 ng/mL. The patient was hospitalized for 34 days, of which 27 were in ICU (Figure 1).

## Methods

Cholinesterase levels were measured using petrochemistry products, Ortho Medical Equipment Trade (China) Co., Ltd. slides, and the Medical Device of the PRC Registration Certificate NO.: 20212402803. Dry tablets (rate method) and compound calibrator for cholinesterase were determined by cholinesterase on a vitros5600 automatic biochemical immunoanalyzer (VITROS Chemistry Products, Calibrator kit 6). The reportable range of measurement was 200–12,500 U/L.

LC-MS was used to detect atropine,PAM,phorate and its metabolites. Ultra-high-pressure liquid chromatography-tandem mass spectrometry on a Sciex Triple TOFTM 5600+ mass spectrometer was used for toxic metabolites terminology screening. Ultra-high-pressure liquid chromatography-tandem mass spectrometry on a Sciextriplequad5500 mass spectrometer was used to detect target analytes quantitatively. Liquid chromatographic separations were performed with a Kinetex C18 column (2.1*50 mm,2.6 μm) from Phenomenex (Torrance, California, United States).Data were processed using the Xcalibur software (version2.0.7) from ThermoFisher.

## Results

Within the 2 weeks, the lowest cholinesterase levels were between 200 and 300 U/L. After 2 weeks, the level of cholinesterase began to rise again. After another 2 weeks, the level of cholinesterase began to rise obviously. Changes in WBC were mainly due to tracheotomy and infection during treatment (Table 1.).

**TABLE 1:** Results of laboratory assistant examination of patient.

Date	1	3	7	14	28
ACHE(u/L)	268	200	200	233	3621
WBC(10^9^/L)	14	7.21	10.62	17.89	8.31
NEUT (%)	89.8	79.11	80.64	87.14	76.7
RBC(10^12^/L)	4.75	4.09	3.77	3.41	3.1
HGB (g/L)	142	125	114	103	106
HCT	0.428	0.381	0.347	0.331	0.334
PLT (10^9^/L)	192	175	209	500	344
ALT (u/L)	76	27	13	231	20
AST (u/L)	79	16	17	129	16
ALB (g/L)	40	35	32	31	35
BUN(mmol/L)	7	6.8	6.6	5.6	5.5
Cr (umol/L)	55	65	50	45	40
UA (umol/L)	347	242	79	73	88
K (mmol/L)	4.7	4.23	4.46	4.83	4.6
Na (mmol/L)	135	135	135	139	136
Cl (mmol/L)	111	102	104	100	99
Ca (mmol/L)	2	1.97	2.01	2.12	2.2
GLU (mmol/L)	5.9	8.53	11.8	10.4	7.6
PT(s)	12.9	12.1	12.8	11.5	11.6
APTT(s)	147.7	27	26.6	29	28
INR	1.14	1.07	1.03	1.02	1.02
BNP(pg/mL)	5	12.5	5	—	—
TNI(ng/mL)	<0.05	<0.05	<0.05	—	—
MYO(ng/mL)	185	39	275	—	—
CKMB(ng/mL)	1.9	<1	<1	—	—
DD2 (mg/L)	1810	651	2720	—	—

**FIGURE 2:**
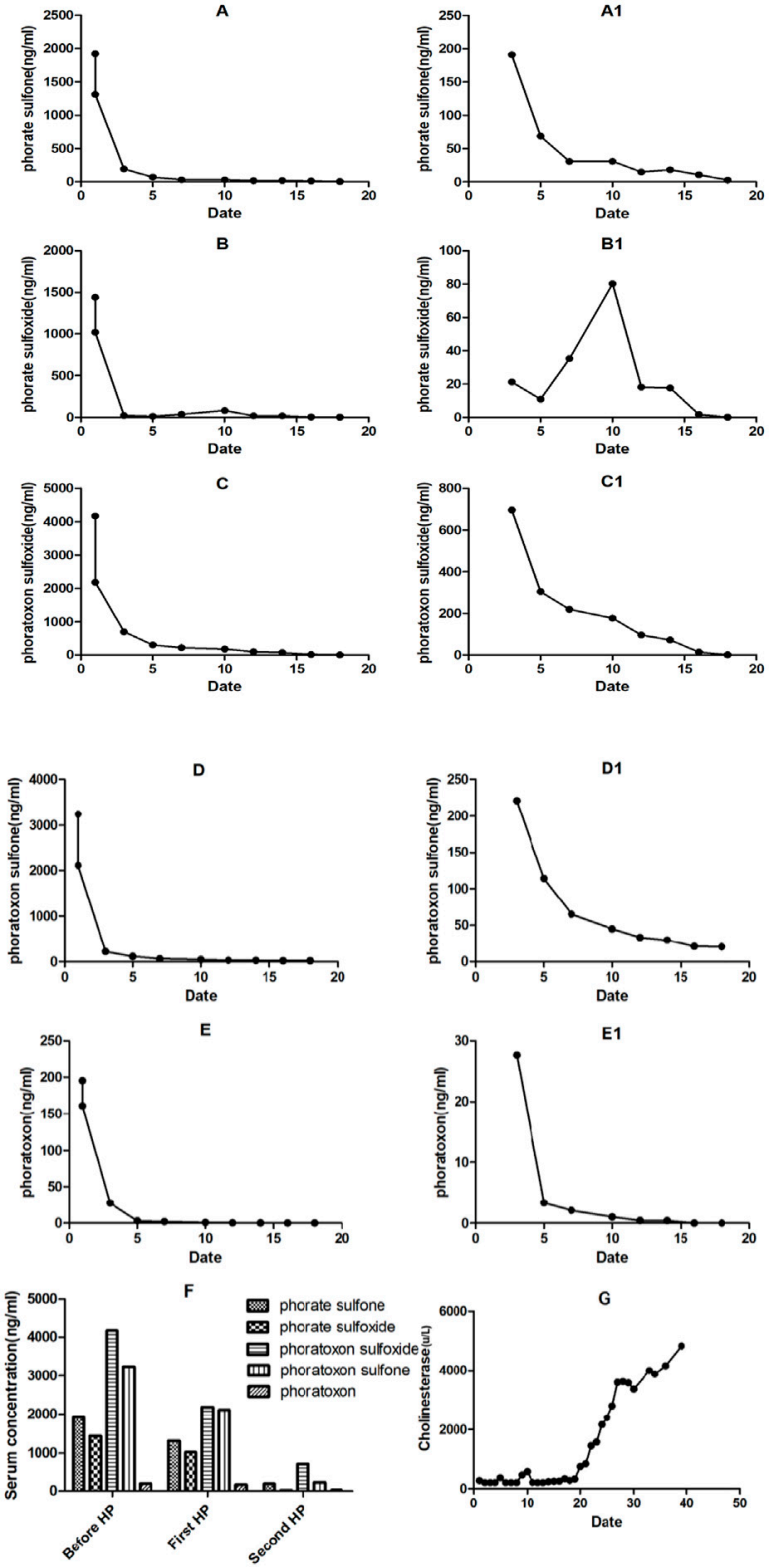
Changes in serum **(A–F)** and urine **(A1–E1)** levels of phorate metabolites and cholinesterase after HP treatment **(G)**.

The blood test before the first HP did not detect phosphates. The levels of various phosphate metabolites are shown in Figure 2. Phoratoxon sulfoxide had the highest concentration, followed by phoratoxon sulfone, phosphate sulfone, phosphate sulfoxide, and phoratoxon (McLeod et al., 1969). After the first HP treatment and subsequent daily HP treatments, phorate was not detected, while the concentration of phorate metabolites significantly fell with treatment. Notably, phorate sulfone and phorate sulfoxide serum levels rose between the seventh and 10th day of admission due to increased toxic metabolites terminology levels after the patient’s family rejected HP treatment. Because the change range of metabolite concentration is very large, it is more intuitively presented in the corresponding Figures 2A1–E1. From the seventh-10th day, when the patient’s family rejected HP, catalytic production of phosphate was observed, accompanied by a significant rise in phorate sulfoxide levels (Figure 2B1) and a slight rise in phorate sulfone levels (Figure 2A1). The serum levels of phoratoxon sulfoxide, phoratoxon sulfone, and phoratoxon gradually fell in this period (Table 2). Relative to before treatment, serum levels of phorate metabolites significantly fell after the first HP and second HP treatment (Figure 2F). Serum cholinesterase levels did not significantly change in 20 days, after which they began to rise and returned to about 50% of the normal value after 30 days.According to the guidelines for the diagnosis and treatment of organophosphorus poisoning, clinical cure can be confirmed and the patient can be discharged when the cholinesterase level rises to 50% of the normal value (Thiermann et al., 1997; Clinical Expert Consensus on the, 2016).

**TABLE 2:** Changes of serum concentration of metabolites of phorate.

Date	phorate sulfone	phorate sulfoxide	phoratoxon sulfoxide	phoratoxon sulfone	phoratoxon	phorate
1(Before HP)	1920	1440	4170	3240	195	—
1 (After HP)	1310	1020	2180	2110	161	—
3	191	21.2	696	221	27.7	—
5	68.9	10.9	304	114	3.33	—
7	30.7	35.2	219	65.2	2.10	—
10	31	80.2	177	44.9	1.01	—
12	14.8	18.1	96.2	33.3	0.433	—
14	18.2	17.6	72.3	29.4	0.415	—
16	10.8	1.67	14.5	20.9	0	—
18	2.71	0	0.743	20.3	0	—

Note: as phorate is not detected in serum samples, no results are given.Serum concentration unit of metabolite:ng/mL.

**FIGURE 3:**
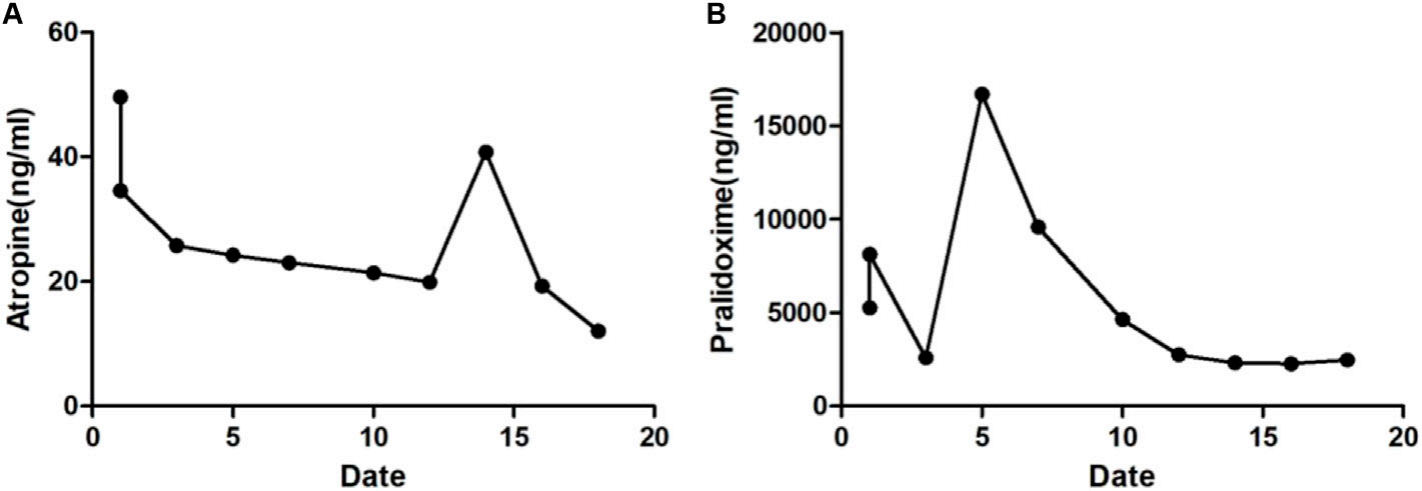
The levels of serum atropine **(A)** and PAM **(B)**.

Figure 3 show serum atropine and PAM levels during treatment. The long-term daily use of PAM maintained the effective treatment concentration of atropine and PAM (Clinical Expert Consensus on the, 2016).

## Discussion

Phorate (phosphorodithioic acid O, O-diethyl S-[(ethylthio)methyl] ester, C_7_H_17_O_2_PS_3_) is a transparent, slightly smelly oily liquid. Phorate emulsion (60%) contains, in addition to the pesticide itself, 20%–30% toluene and xylene as solvents, 10%–15% surfactants such as alkylphenol ethoxylates and calcium dodecylbenzenesulfonate, 5%–10% ethylene glycol as an antifreeze agent, and trace amounts of odorants for warning purposes (Sun et al., 2017). All of the aforementioned substances are of low toxicity and have no impact on the determination of the concentrations of cholinesterase, PAM, phorate, and its metabolites studied in this case (National Center for Biotechnology Information, 2025a; National Center for Biotechnology Information, 2025f; National Center for Biotechnology Information, 2025g; National Center for Biotechnology Information, 2025b; National Center for Biotechnology Information, 2025c). The routes for acute phorate poisoning include oral ingestion, inhalation, or contact with skin and mucosa. Clinical signs of acute phorate poisoning include headache, dizziness, anorexia, nausea, vomiting, abdominal pain, diarrhea, salivation, pupil reduction, increased respiratory secretion, hyperhidrosis, and fascicular tremor. In severe cases, it can cause pulmonary edema, cerebral edema, coma, and respiratory paralysis. Some cases may cause heart, liver, and kidney damage (Farooqui et al., 2020). Phorate poisoning reduces blood cholinesterase.

Once absorbed, phorate is rapidly distributed throughout the body owing to its liposolubility (logP = 3.71) and is mainly distributed to fatty tissues, including the brain, liver, and fat. It is then oxidized into phoratoxon, phoratoxon sulfoxide and phoratoxon sulfone by P450 monooxygenase and flavin adenine dinucleotide monooxygenase (Cheropkina et al., 2023; Moyer et al., 2018a). Phorate is mainly metabolized in the live. The toxicity of these oxidation products is higher than that of phorate itself (Cheropkina et al., 2023; Moyer et al., 2018a), resulting in increased acetylcholine amounts at the cellular and subcellular levels. Phorate is also directly catalyzed into phorate sulfoxide and phorate sulfone (Cheropkina et al., 2023; Moyer et al., 2018a; Hodgson et al., 1995). The cholinesterase inhibition by phorate and phorate sulfoxide is weak (IC50 = 3100 and 1500 μM, respectively). Phorate sulfone (IC50 = 40 μM) is about 80 times more toxic than phorate. Relative to phorate, its three oxidative metabolites are 1000 times more toxic (phoratoxon, phoratoxon sulfoxide, and phoratoxon sulfone IC50 = 3, 0.9, and 0.5 μM, respectively) (Kovarova et al., 2013). Unmetabolized phorate is no longer detectable in blood 24 h after ingestion and only exists in metabolites. Although the time between ingestion and the first blood analysis before HP was about 4 h, we did not detect unmetabolized phorate. Highlighting the swift metabolization of phorate upon absorption into three oxidation products and two catalytic products.

The blood test before the first HP revealed that the phorate metabolite with the highest concentration was phoratoxon sulfoxide, followed by phoratoxon sulfone, phorate sulfone, phorate sulfoxide, and phoratoxon. Consistent with published reports, phorate was undetectable after the initial HP and subsequent daily HP treatments but existed in the form of its metabolites. Phorate levels decreased with treatment. Notably, between the seventh and 10th day, when HP treatment was stopped, the serum levels of the phorate catalytic products, phorate sulfone and phorate sulfoxide, rose. This may be attributable to a continuous release of toxic metabolites accumulated in the liver, brain, fat and other organs. However, the serum levels of phoratoxon sulfoxide, phoratoxon sulfone, and phoratoxon did not rise. Between the seventh and 10th day, when HP treatment was stopped, the levels of these three poisons continued to fall gradually. Even without HP treatment, the three phorate oxidative metabolites were continuously metabolized out of the body.

However, the metabolism of the two phorate catalytic products may be too slow, causing the amount released to blood circulation to exceed the concentration cleared from the body. Hence causing the blood levels of the two catalytic products to rebound. The precise underlying mechanism warrants investigation. However, our data show that toxic metabolites continuously release to blood circulation. Thus, without continuous HP, atropine, and PAM, the patient’s phorate poisoning would have rebounded. Therefore, we hypothesize that if the treatment is inadequate, including insufficient blood purification or its suspension, or insufficient or premature discontinuation of atropine and PAM, relapse of the condition may occur. The toxic metabolites potentially responsible for the relapse could be the three oxidative metabolites of phorate or phorate sulfone. Given the toxic effects of phorate sulfone and its sustained release in the body, it is more likely to contribute to the recurrence of symptoms. In contrast, phorate sulfoxide has a weaker inhibitory effect on cholinesterase. Although it also exerts a sustained release effect in the body, the likelihood of exacerbating the condition is relatively low.

In organophosphorus poisoning, cholinesterase combined with organophosphorus cannot effectively degrade acetylcholine. Thus, acetylcholine accumulates in neuromuscular joints, causing muscarinic symptoms (M-like symptoms) and nicotinic symptoms (N-like symptoms) (Hulse et al., 2019). Atropine can competitively antagonize acetylcholine activity on cholinergic receptors, effectively relieving these symptoms. Figure G shows that the change in cholinesterase serum levels is very slow and was not significantly altered in 20 days, after which the patient’s serum cholinesterase levels began to recover, reaching 50% of normal levels in 30 days. Even if recovery produces small amounts of cholinesterase, it is bound by the toxic metabolites being continuously released in the organs. Thus, after 20 days, the serum toxic metabolites were reduced enough for the patient’s cholinesterase to begin accumulating. Hence the fast cholinesterase recovery after 20 days (Figure 1G) (Thiermann et al., 2007). This observation challenges previous assumptions of cholinesterase ageing, which was believed to explain the prolonged low levels of cholinesterase. In this case, the sustained low levels of cholinesterase may suggest the continuous toxic effects of phorate oxidative metabolites and the sustained release of catalytic products within the organs.

Atropine dosage for severe organophosphorus poisoning has been debated for a long time because atropine is also toxic (LD50 = 100 mg/d) (Eddleston et al., 2008; Beards et al., 1994). In the first 15 days after admission, the patient was treated with atropine at 5–10 mg/h (about 120–240 mg/D). After effective atropinization, the dosage was maintained continuously. Although large atropine doses cannot relieve the patient’s symptoms, they can speed up the heart rate, improve myocardial oxygen consumption, and cause systemic parasympathetic extreme inhibition (Thiermann et al., 2010). To ensure the lowest dose of atropine in the treatment, we monitored serum atropine levels. Previous studies and guidelines recommend using PAM for short durations (Eddleston et al., 2008; Worek et al., 1997). PAM is a cholinesterase reactivator, whose quaternary ammonium group can tend to bind to the cationic site of phosphorylated cholinesterase that has lost its activity and is bound to organic phosphorus. Its nucleophilic group can directly bind to the phosphorylated group of cholinesterase and then jointly detach from cholinesterase, restoring the original state of cholinesterase (Eddleston et al., 2004). However, because the redistribution and re-release of phorate metabolites into the blood can regenerate phosphocholinesterase, we used PAM for a long time while monitoring its serum level. Moyer et al. found that the aging of AChE inhibited by phorate was very slow, and no significant aging was observed within 48 h. The therapeutic window for oxime administration following exposure to phorate is not limited by aging. Therefore, we used PAM for a total of 15 days (Moyer et al., 2018b). Bloch-Shilderman et al. describes exactly this phenomenon of slow release of the agent (from a dermal depot) to blood circulation, thus continuously inhibiting new *de novo* synthetized ACHE (Bloch-Shilderman et al., 2023).

Past research has suggested that the therapeutic dosage of atropine is very close to its toxic dose, and excessive use may lead to atropine toxicity. Additionally, it has been thought that PAM is best used for short periods. However, by dynamically monitoring the concentrations of toxic substances, cholinesterase, atropine, and PAM, we achieved precise treatment, avoiding atropine toxicity while optimizing the effects of PAM.

In the selection of sedative-hypnotic drugs, we chose midazolam and dexmedetomidine. The combined use of these two drugs can effectively counteract mental symptoms such as agitation caused by atropine. During the medication period, we implemented a daily awakening plan, stopping all sedative drug infusions between 7:30 and 7:50 every morning. Thirty minutes after drug withdrawal, we assessed the level of consciousness and simple instructions, and adjusted the dosage based on the RASS score, with the goal of maintaining a RASS score between −2 and −3 (Cornelissen et al., 2024). However, in the early stage, the patient was obviously agitated and uncooperative in treatment. Therefore, the two drugs were used for a total of 23 days. After the cholinesterase level significantly increased and the atropine dosage was reduced, the patient’s agitation was alleviated, and the patient became cooperative in treatment. The drugs were then gradually withdrawn.

## Conclusion

The clinical management of severe organophosphorus poisoning remains particularly challenging. A comprehensive treatment strategy combining anticholinergic medications, oxime-based cholinesterase reactivators, and extracorporeal blood purification techniques is essential for optimizing patient outcomes. Following absorption, the parent toxin undergoes extensive hepatic biotransformation, generating multiple bioactive metabolites through oxidative and enzymatic pathways. These metabolites collectively contribute to the systemic toxic manifestations observed in severe poisoning cases.

Blood purification interventions demonstrate significant efficacy in reducing circulating metabolite concentrations. However, sustained therapy appears critical for maintaining metabolic equilibrium, as premature discontinuation may result in complex alterations of metabolite profiles. Specifically, oxidative metabolites tend to decrease over time while certain enzymatic breakdown products may paradoxically increase, highlighting the dynamic nature of toxicokinetic processes during recovery.

Functional recovery of cholinesterase activity occurs gradually, often requiring prolonged clinical monitoring even after acute symptom resolution. These observations underscore the need for individualized treatment protocols that address both the pharmacodynamic and pharmacokinetic aspects of organophosphorus poisoning.

Of course, this study has limitations. While we dynamically observed and monitored the concentrations of the toxic metabolites and medications, this is ultimately a case report, and the features discovered during treatment need to be validated by more clinical cases and basic research in the future.

## Data Availability

The original contributions presented in the study are included in the article/Supplementary Material, further inquiries can be directed to the corresponding author.
